# Review of the genus *Agria* (Diptera, Sarcophagidae) from China

**DOI:** 10.3897/zookeys.310.5346

**Published:** 2013-06-19

**Authors:** Ming Zhang, Yi-ou Chen, Thomas Pape, Dong Zhang

**Affiliations:** 1 College of Nature Conservation, Beijing Forestry University, Beijing 100083, China; 2 Natural History Museum of Denmark, University of Copenhagen, Universitetsparken 15, DK–2100 Copenhagen, Denmark

**Keywords:** Paramacronychiinae, *Agria*, coeloconic sensilla, new record, China

## Abstract

*Agria mihalyii* (Rohdendorf and Verves, 1978) is recorded from China for the first time, and both sexes are thoroughly documented using a combination of illustrations, photographs and scanning electron microscopy images. The generic affiliation is corroborated from an expanded definition of genus *Agria* Robineau-Desvoidy, 1830, and a key to males of the two known species from China is provided. The distribution of coeloconic sensilla on the male pre- and postgonite are shown to possess significant diagnostic and phylogenetic information in this genus.

## Introduction

The genus *Agria* Robineau-Desvoidy is a small genus of the subfamily Paramacronychiinae (Sarcophagidae), occurring in the Holarctic region and comprising eight species worldwide (Pape 1987, 1996, 1998, Fan and Pape 1996). The larvae are parasitoids of last instar larvae and pupae of Lepidoptera, occasionally also attacking sawflies (Roback 1954, Pape 1987, Kuhlmann 1995). Pape (1987, 1992, 1998) published a series of comprehensive taxonomic studies including this genus, which formed the basis for further research on this genus.

Before the present contribution, only *Agria affinis* (Fallén, 1817) was known from China (Fan and Pape 1996). Chao and Zhang (1988) described *Agria xiangchengensis* from Xiangcheng, Sichuan, but the species does not possess any of the features usually considered diagnostic for species of *Agria*, which led Pape (1996) to catalogue it under “Unplaced species-group taxa of Paramacronychiinae”, and it was subsequently made the type species of a monotypic genus *Mimagria* by Verves (2001). While checking a series of *Agria* specimens from Beijing and Shanxi, we found one additional Chinese species, *Agria mihalyii* (Rohdendorf and Verves, 1978). The primary aims of this article are to review the genus *Agria* from China; to redescribe the newly recorded species; to provide detailed documentation through illustrations, photographs and scanning electron microscopy images of *Agria affinis* and *Agria mihalyii*; and to refine the scientific definition of the genus. A key to the known species of *Agria* from China is also provided.

## Materials and methods

The specimens examined were collected by sweeping from brushwood in mountainous regions and are deposited in the Museum of Beijing Forestry University, Beijing, China.

Methods for the preparation of terminalia, illustrations, photographs and scanning electron microscopy images follow Zhang et al. (2013).

Terminology of male morphology and terminalia follows McAlpine (1981) and Giroux et al. (2010), except for the term ‘lateral sclerotization’, which follows Pape (1992). Distributional data is taken from Pape (1996).

## Taxonomic account

### Agria affinis

(Fallén, 1817)

http://species-id.net/wiki/Agria_affinis

Figs 1 and 7

Musca affinis Fallén, 1817: 237.
Agria punctata : Robineau-Desvoidy 1830: 377; Pape 1987: 85; Fan et al. 1992: 613.
Agria affinis : Verves 1982: 273; Pape 1996: 158; Fan and Pape 1996: 244.

#### Material examined.

China: Beijing: 1 ♂, Xiaolongmen, 39°57'50"N, 115°28'26"E, 1100 m, 6.VII.2009, Coll. R. Bi and F. Li; 1 ♂, Mt. Songshan, 40°30'00"N, 115°49'12"E, 800−1000 m, 30.V.2012, Coll. Y.O. Chen; 1 ♂, Mt. Songshan, 40°30'00"N, 115°49'12"E, 800−1000 m, 30.V.2012, [collector unknown].

#### Distribution.

China (Beijing, Qinghai, Xinjiang); Mongolia; Kazakhstan; Kyrgyzstan; common throughout Europe.

**Figure 1.**
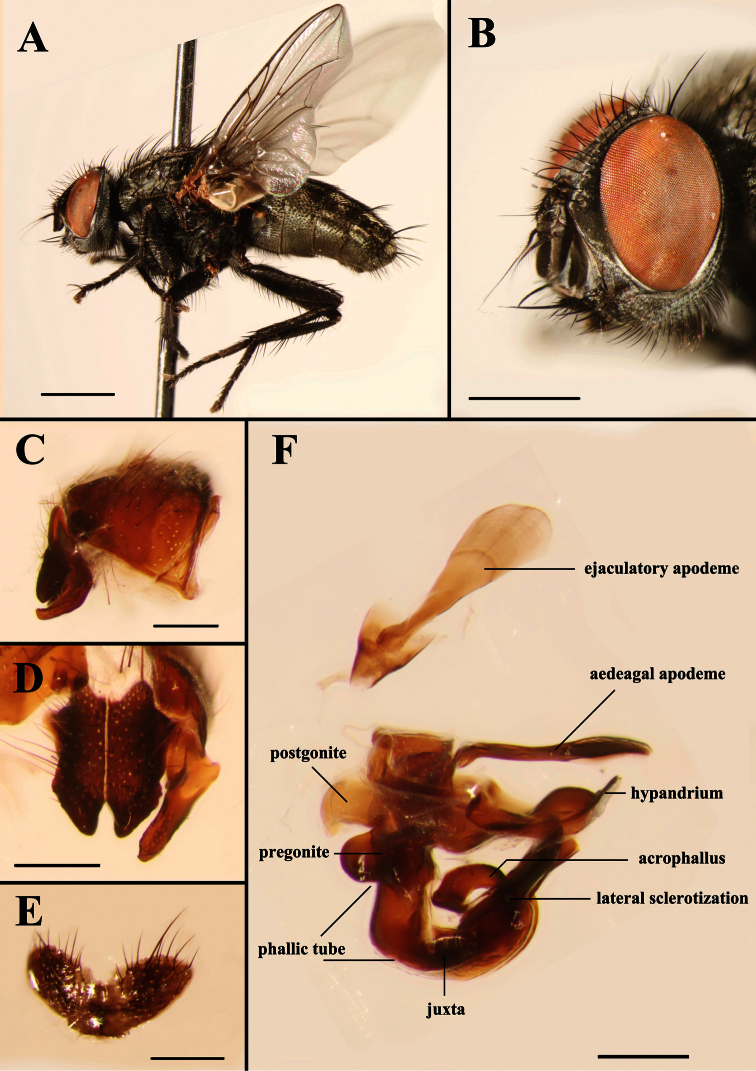
Light micrographs of the male *Agria affinis* (Fallén, 1817). **A** Habitus, lateral view **B** Head, anterolateral view **C** Terminalia, epandrium, surstylus and cercus, lateral view **D** Surstylus and cerci, dorsal view **E** Sternite 5, ventral view **F** Genitalia, lateral view. Scale bars: **A**= 2.00 mm, **B**= 1.00 mm, **C−F**= 0.25 mm.

### Agria mihalyii

(Rohdendorf and Verves, 1978)

http://species-id.net/wiki/Agria_mihalyii

**Figure 2.**
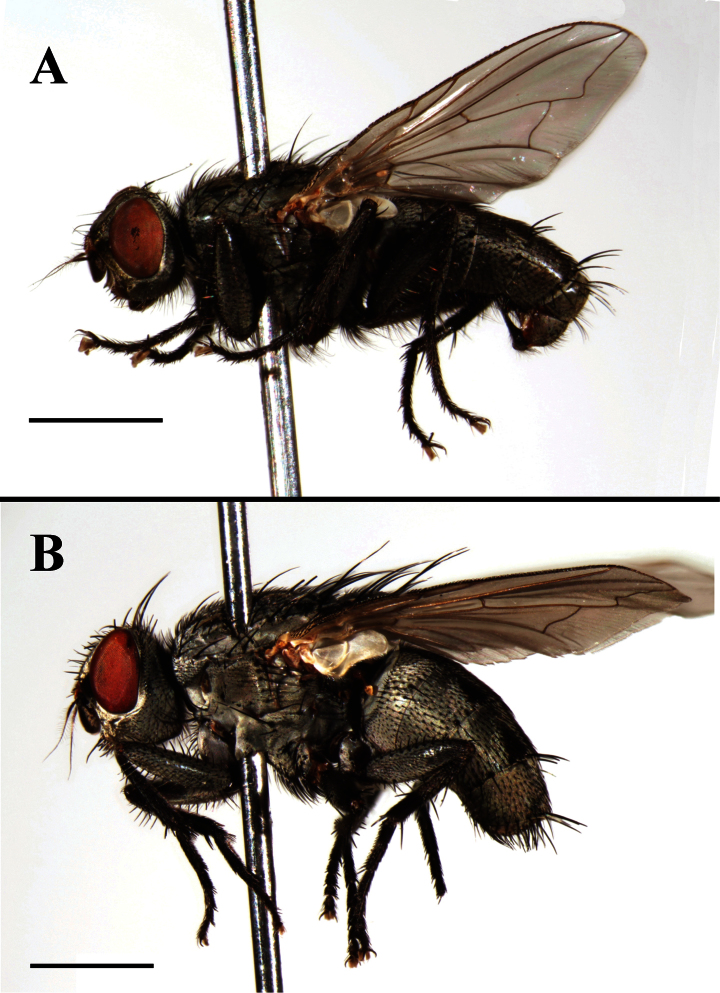
*Agria mihalyii* (Rohdendorf and Verves, 1978). **A** Male habitus, lateral view **B** Female habitus, lateral view. Scale bars: A and B= 2.00 mm.

Figs 2 [Fig F3] [Fig F4] − 6 , 8 and 9

Angiometopa mihalyii Rohdendorf and Verves 1978: 247.
Angiometopa mihalyii : Verves 1981: 198, 1982: 279.
Agria mihalyii : Pape 1992: 309, 1996: 159

#### Redescription.

MALE. Body length 7.8−10.4 mm. Eyes bare. Fronto-orbital and parafacial plates black with grey pollinosity; postocular strip bare and with silvery grey pollinosity; parafacial plate and fronto-orbital plate with rows of fine bristles. Frontal vitta black, 2.10 × as broad as fronto-orbital plate at the narrowest point; frons at vertex 0.30 × head width; frontal row of 9−12 strong bristles; outer vertical bristle not differentiated from postocular bristles, upper orbital bristle one. One pair of strong ocellar bristles. Gena ground color black, with sparse and short black bristles and silvery grey pollinosity, height 0.47 × eye height in lateral view. Antenna slightly reddish basally, otherwise blackish, not reaching the level of vibrissal insertion, first flagellomere 1.70 × as long as pedicel; arista black brown, short plumose in basal 3/5–2/3. Palpus orange. Thorax ground color black; scutum with three black dorsal vittae. Chaetotaxy: acrostichals 0+1, dorsocentrals 2(3)+3, intra-alars 1(0)+2(3), supra-alars 2, postpronotals 3, postalars 3 or 4, notopleurals 2, scutellum with 1 apical, 1 subapical, 1 basal and 1 discal bristles, with or without prebasal bristle. Pleuron with meropleurals 8−10, katepisternal bristles 2(3)+1, prosternum and metasternum bare, proepisternum bare, proepimeron in lower part with fine bristles, postalar wall bare or with fine bristles. Wing hyaline; subcostal sclerite and basicosta yellow, bare; tegula dark yellow, with black setulae; costal spine not differentiated, several dorsal black bristles at node of R_4+5_-R_2+3_. Legs dark; fore femur with one strong row of posterior bristles, and with long and dense bristles along anteroventral, ventral and posteroventral margins, fore tibia with four anterodorsal and one posterior bristles; mid femur with two anterior and two posterior bristles, and distal 1/3 with short ventral comb-like posteroventral bristles, mid tibia with two or three anterior and one or two hair-like posterior bristles; hind femur with one row of anterodorsal bristles, and with long and dense bristles along anteroventral, ventral and posteroventral margins, hind tibia with one posterodorsal bristle, one row of anteroventral bristles (7 or 8) and one row of anterodorsal bristles (3 or 4). Abdomen long oval with densely grey pollinosity; tergites each with three distinct black spots; tergite 3 without median marginal bristles, tergite 4 with one pair of median marginal bristles, tergite 5 with strong marginal bristles, tergites 7+8 form a hump-shaped structure, epandrium brownish black, sternites 1−4 with long and dense bristles. Terminalia: Cercus tapering and pointed distally, basal 1/3 with long dense bristles; surstylus long and with oval rounded tip in lateral view (Fig. 5A). Ejaculatory apodeme large (Figs 4 and 8B). Pregonite broad, longer than postgonite, with some fine bristles on the basal part (Fig. 8C), and distal half perpendicular to basal half (Figs 4, 8A, 8C, 9A and 9D); postgonite broad with curved tip and a strong bristle proximally on anterior margin, six coeloconic sensilla (2.10 µm in height, 1.68 µm in width at the base and 1.20 µm at the middle, and originating from a cuticular ring inside a shallow depression) distributed on distal half (Figs 8E and 8F); juxta very large and shell-shaped, apically with a pair of slanting processes covering most of the acrophallus in lateral view (Figs 4, 8A and 9A−C); phallic tube broad, with the dorsal part dark; acrophallus very broad basally, the distal part strongly tapering and recurving between the juxta (Figs 4, 9B and 9C); lateral sclerotizations short, with a serrated distal margin (Figs 4 and 9C).

FEMALE. Body length 7.0−9.0 mm. Frons at vertex 0.40 × as broad as head width; frontal row of 9 or 10 bristles; outer vertical bristle differentiated from postocular bristles, proclinate orbital bristles two. Gena height 0.40 × eye height in lateral view. First flagellomere length 1.40 × as long as pedicel. Thorax chaetotaxy: acrostichals 0+2, intra-alars 1+2. Fore femur with one posterior, one posterodorsal and one posteroventral rows of bristles; mid femur with short and sparse ventral bristles, without apical comb-like posteroventral bristles, mid tibia with two posterodorsal and two posterior bristles, one strong ventral bristle; hind tibia with two or three posterodorsal bristles, one anteroventral bristle. Abdomen oval; tergites 5 and 6 entire, tergite 7 membranous like with several bristles on the anterior margin, tergite 8 divided into two plates and each with two strong bristles (Fig. 6E); sternites 1−6 without long and dense bristles (Fig. 6A); epiproct as a single setose plate, hypoproct and sternite 8 sclerotized (Fig. 6E). Other morphological characteristics are the same as for the male.

#### Material examined.

China: Shanxi: 3 ♂♂, Tianzhen county, 40°24'00"N, 114°6'00"E, 1600−1700 m, 24.V.1987, Coll. M.F. Wang; 1 ♂, Yuxian county, Mt. Zangshan, 38°6'00"N, 113°24'00"E, 900−1000 m, 23.VI.1999, Coll. M.F. Wang. Beijing: 1 ♂, Mt. Songshan, 40°30'00"N, 115°49'12"E, 800−1000 m, 5.VII.2008, [collector unknown]; 1 ♂, Mt. Songshan, 40°30'00"N, 115°49'12"E, 800−1000 m, 5.VI.2009, [collector unknown]; 1 ♂, Mt. Songshan, Daxigou, 40°31'30"N, 115°46'19"E, 1200 m, 25.VII.2009, Coll. D. Zhang; 1 ♀, Mt. Songshan, Changyugou, 40°30'00"N, 115°48'57"E, 800 m, 28.VII.2009, Coll. D. Zhang; 2 ♂♂, Mt. Songshan, 40°30'00"N, 115°49'12"E, 800−1000 m, 29.VII.2010, [collector unknown];1 ♂, Mt. Songshan, 40°30'00"N, 115°49'12"E, 800−1000 m, 30.V.2012, Coll. Y.O. Chen.

#### Distribution.

China (Beijing, Shanxi); Mongolia; North Korea; Russia (East Siberia, Far East, West Siberia); Ukraine.

**Figure 3. F3:**
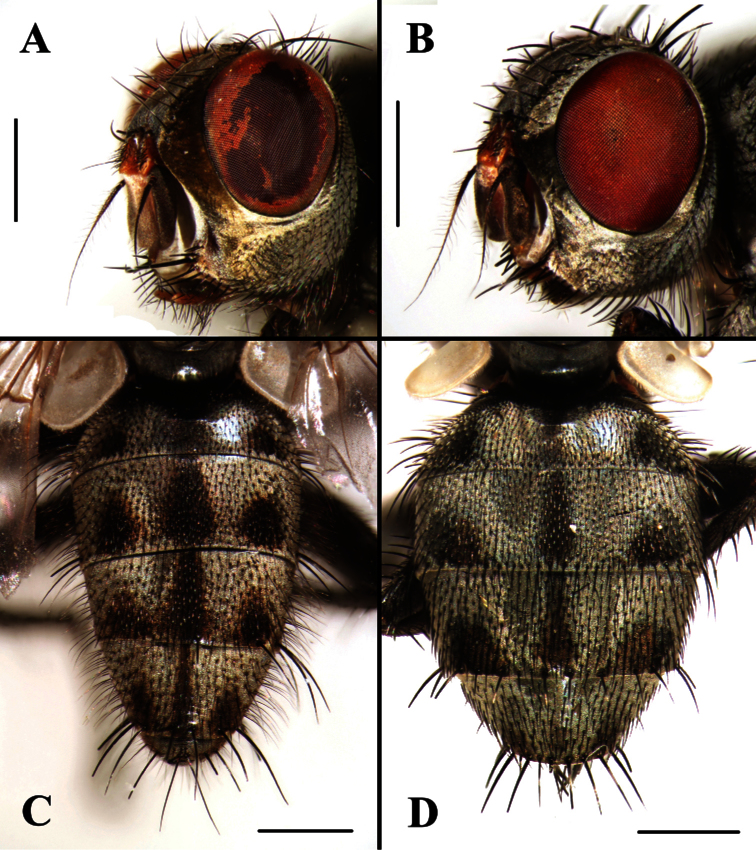
*Agria mihalyii* (Rohdendorf and Verves, 1978). **A** Male head, left anterolateral view **B** Female head, left anterolateral view **C** Male abdomen, dorsal view **D** Female abdomen, dorsal view. Scale bars: **A−D**= 1.00 mm.

**Figure 4. F4:**
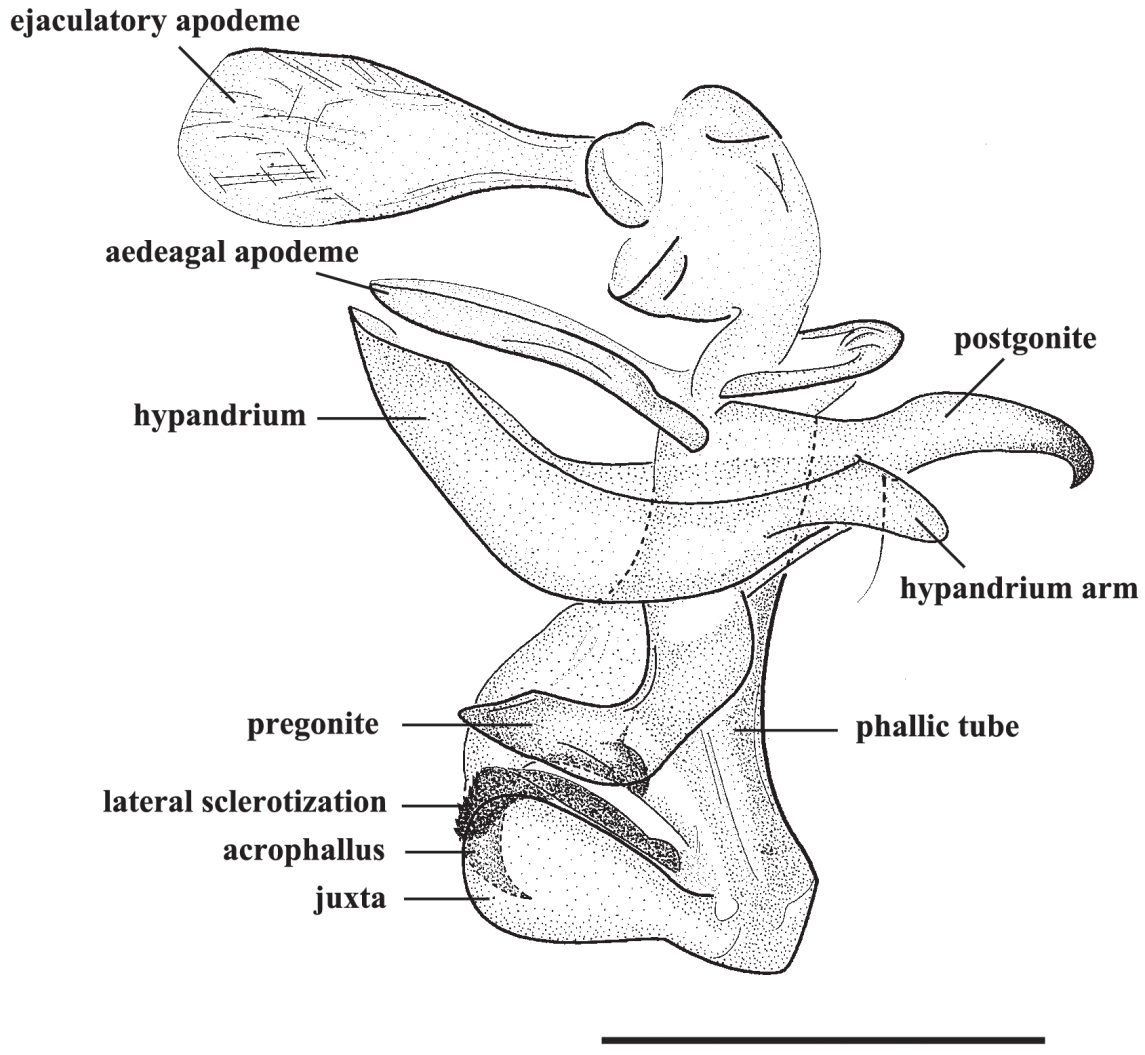
*Agria mihalyii* (Rohdendorf and Verves, 1978). Male, genitalia, lateral view. Scale bar = 0.50 mm.

**Figure 5.**
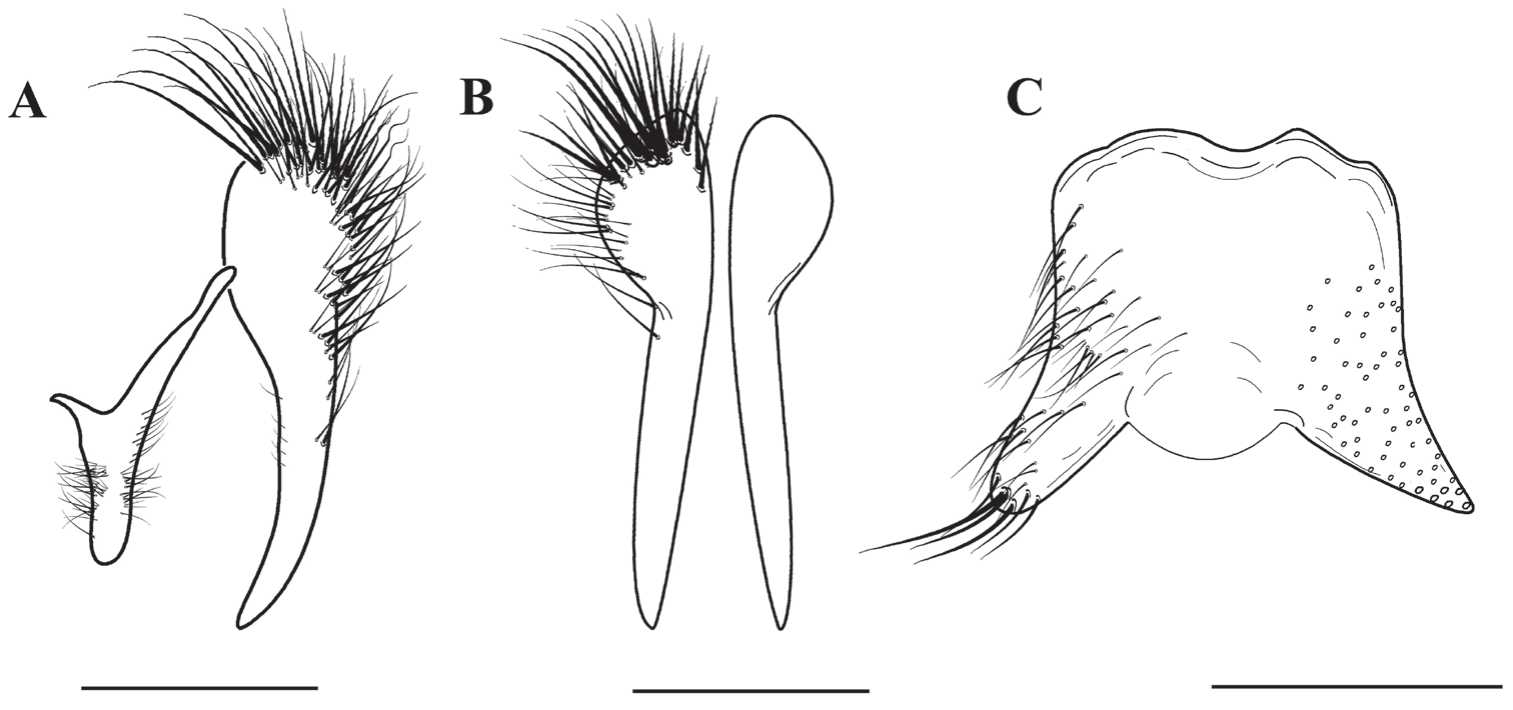
*Agria mihalyii* (Rohdendorf and Verves, 1978). Male, terminalia. **A** Cercus and surstylus, lateral view **B** Cerci, dorsal view **C** Sternite 5, ventral view. Scale bars: **A−C** = 0.50 mm.

**Figure 6.**
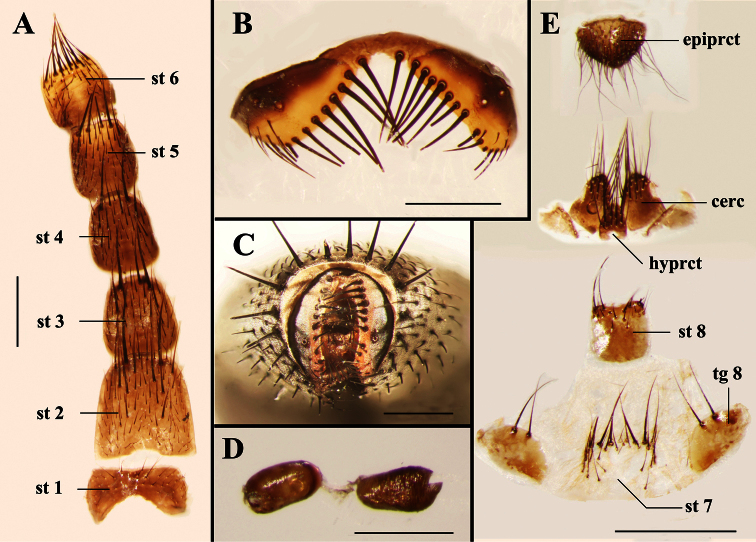
Light micrographs of the female terminalia of *Agria mihalyii* (Rohdendorf and Verves, 1978). **A** Sternites 1−6, ventral view **B** Tergite 6, dorsal view **C** Terminalia, posterior view **D** Spermathecae **E** Terminalia, ventral view. Scale bars: **A−C** and **E**= 0.50 mm, **D**= 0.25 mm. Abbreviations: cercus (cerc); epiproct (epiprct); hypoproct (hyprct); sternite (st); tergite (tg).

**Figure 7.**
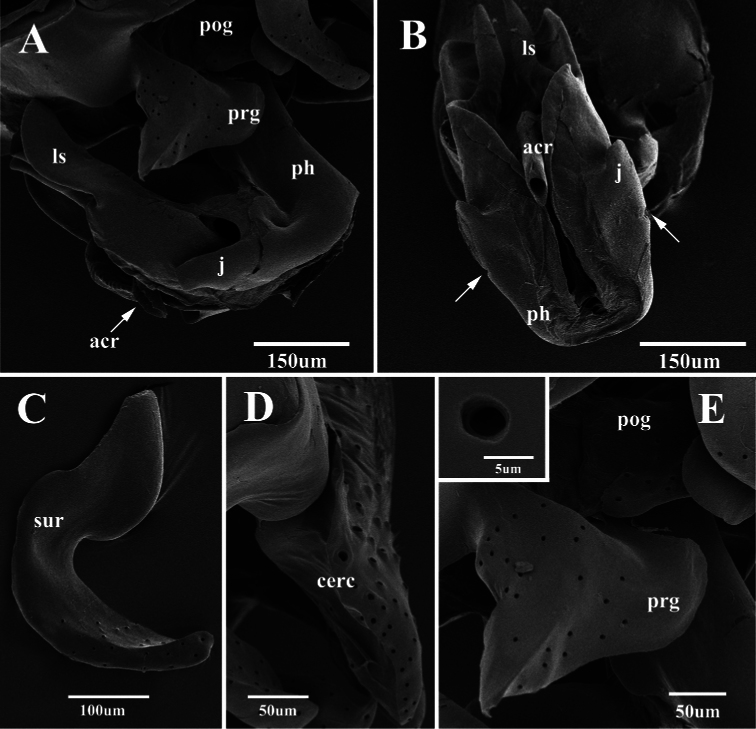
Scanning electron micrographs of the male genitalia of *Agria affinis* (Fallén, 1817). **A** Lateral view of the genitalia **B** Ventral view of distiphallus, arrows show the dividing line between phallic tube and juxta **C** Surstylus, lateral view **D** Cercus, lateral view **E** Pregonite and base of postgonite enlarged view, with inset showing highly enlarged view of one of the sockets from a coeloconic sensilla. Abbreviations: acrophallus (acr); cercus (cerc); juxta (j); lateral sclerotization (ls); phallic tube (ph); postgonite (pog); pregonite (prg); surstylus (sur).

**Figure 8.**
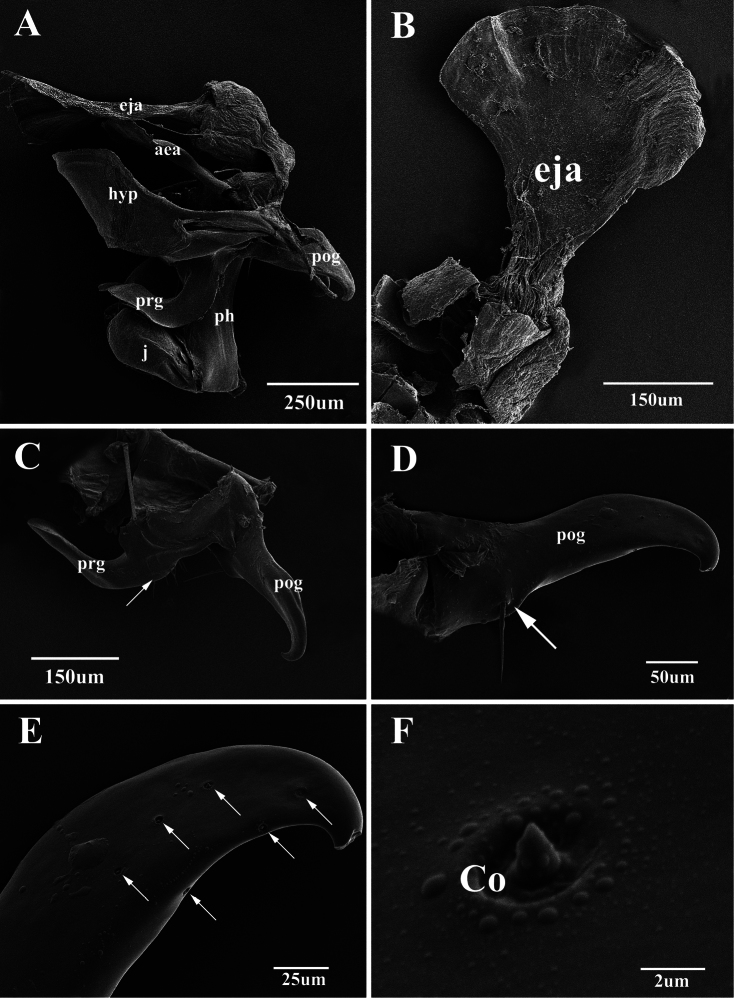
Scanning electron micrographs of the male genitalia of *Agria mihalyii* (Rohdendorf and Verves, 1978). **A** Lateral view of the entire genitalia **B** Ejaculatory apodeme **C** Pregonite and postgonite, the former with some fine bristles at the dorso-basal edge (arrow) **D** Postgonite, with one well developed bristle (arrow) near base of anterior margin **E** Distal half of postgonite (extreme tip broken), arrows show the distribution of coeloconic sensilla **F** Coeloconic sensilla on the postgonite. Abbreviations: aedeagal apodeme (aea); coeloconic sensillum (Co); ejaculatory apodeme (eja); hypandrium (hyp); juxta (j); phallic tube (ph); postgonite (pog); pregonite (prg).

**Figure 9.**
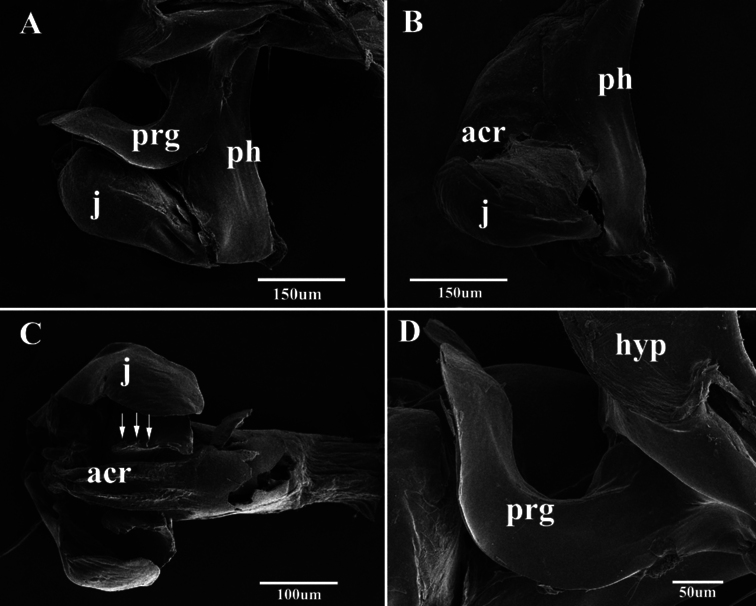
Scanning electron micrographs of the male genitalia of *Agria mihalyii* (Rohdendorf and Verves, 1978). **A** Lateral view of the genitalia **B** Lateral view of distiphallus **C** Acrophallus in anterior view, apex of the lateral sclerotizations with serrated margin (arrows) **D** Pregonite, enlarged view. Abbreviations: acrophallus (acr); hypandrium (hyp); juxta (j); phallic tube (ph); postgonite (pog); pregonite (prg).

### Key to known Chinese species of genus *Agria* Robineau-Desvoidy [males only]

**Table:** 

1	Frons at vertex 0.30 × as broad as head width; antenna slightly reddish basally, palpi orange (Fig. 3A); cerci long, surstylus long and with oval rounded tip (Fig. 5A); pregonite long, distal half perpendicular to basal half (Figs 4 and 9D), postgonite elongate and with a bristle near base of anterior margin (Figs 4, 8D and 8E); lateral sclerotizations short and with serrated distal margin (Figs 4 and 9C); juxta large (Figs 4 and 9A)	*Agria mihalyii* (Rohdendorf and Verves)
–	Frons at vertex 0.16 × as broad as head width; antenna black, palpi at least distally black (Fig. 1B); cerci short, surstylus with apex bent posteriorly (Figs 1C and 1D); pregonite short and strong, almost triangular, postgonite broad and short, without a bristle near base of anterior margin (Fig. 7F); lateral sclerotizations long and without serration (Fig. 1F); juxta small, fused with phallic tube except for an obscure dividing line laterally (Figs 1E, 7A and 7B)	*Agria affinis* (Fallén)

## Discussion

Pape (1998) investigated the phylogenetic relationships of the world genera of Paramacronychiinae but his analysis gave very little phylogenetic resolution among the genera. More novel morphological characters should be added; especially the structures of the male paramacronychiine genitalia need a much more detailed comparative study. The scanning electron microscopy was here utilized to achieve more morphological details of the male genitalia of *Agria*. Two features seem to be particularly valuable in corroborating the monophyly of *Agria*: (1) the shape of the acrophallus, which from a broad base curves back strongly between the juxta, and (2) the pair of lateral sclerotizations originating just distal to the acrophallus and slanting antero-ventrally partly covering the latter.

The present SEM documentation has revealed the presence of coeloconic sensilla on the distal half of the *Agria mihalyii* postgonite (Figs 8E and 8F) and on both pre- and postgonite in *Agria affinis*. This type of sensilla has been proposed to be sensitive to chemo-, thermo-, or hygro-stimulation (Altner et al. 1981, Zacharuk 1985, Blackwell et al. 1992, Cribb 1997), and is usually found on the insect antenna, for example: in Diptera (Sukontason et al. 2004, Wang et al. 2012a, Zhang et al. 2012), Lepidoptera(Hunger and Steinbrecht 1998, Binyameen et al. 2012), Hemiptera (Silva et al. 2010, Wang et al. 2012b), and Hymenoptera (Onagbola and Fadamiro 2008). Only few authors have found sensilla on Diptera terminalia (Hooper et al. 1972, Rossignol and McIver 2005, Ngern-Klun et al. 2007, Chaiwong et al. 2007, 2008), and this is the first explicit record of the presence and distribution of coeloconic sensilla on the gonites in the subfamily Paramacronychiinae. These sensilla may aid in copulation (Rossignol and McIver 2005, Chaiwong et al. 2008), and their distribution may therefore be highly species-specific, as indicated by the marked differences between *Agria affinis* and *Agria mihalyii*. Pape (1992) proposed *Agria mihalyii* to be the sister taxon of the remaining species of *Agria*, with the latter clade supported by two character states: (1) costal spine reduced, and (2) male lower calypter at least partly brownish. The material examined for our study has led us to reinterpret *Agria mihalyii* as having a reduced costal spine, which leaves this character state as phylogenetically uninformative for species-level relationships within *Agria*. However, two other character states appear to support a basal split between *Agria mihalyii* and the remaining species of *Agria*, which all share the following: (a) antero-basal bristle of postgonite reduced; and (b) pregonite with numerous coeloconic sensilla scattered across its surface. The latter character state is documented in Fig. 7E for *Agria affinis* and in Kurahashi (1972, figs 2, 3b, 3f) for *Agria monachae*, *Agria hikosana* and *Agria shinonagai*. The distribution of sensilla and other ultrastructural details of male terminalia in Sarcophagidae obviously is a potentially rich source of phylogenetic data, that still remains to be fully explored.

## Supplementary Material

XML Treatment for Agria affinis

XML Treatment for Agria mihalyii
